# A Report on Hospital Gangrene, Erysipelas, and Pyæma

**Published:** 1863-12

**Authors:** 


A REPORT ON HOSPITAL GANGRENE, ERYSIPELAS AND PYÆMIA, AS OBSERVED IN THE DEPARTMENTS OF THE OHIO AND CUMBERLAND. With Cases Appended. By M. GOLDSMITH, Surgeon U. S. V. Published by permission of the Surgeon-General of U.S.A. Louisville. 1863.

This is a very interesting report or monograph of 95 pages, bound in cloth. The subject discussed is a very important one, and the facts presented in this paper cannot be studied too closely. We have not time or space to review the work in the present number of the EXAMINER, but shall refer to it again soon.

